# MiR-200a inhibits epithelial-mesenchymal transition of pancreatic cancer stem cell

**DOI:** 10.1186/1471-2407-14-85

**Published:** 2014-02-12

**Authors:** Yuhua Lu, Jingjing Lu, Xiaohong Li, Hui Zhu, Xiangjun Fan, Shajun Zhu, Yao Wang, Qingsong Guo, Lei Wang, Yan Huang, Mingyan Zhu, Zhiwei Wang

**Affiliations:** 1 Surgical comprehensive laboratory, Affiliated Hospital of Nantong University, Nantong, Jiangsu Province 226001, P. R. China; 2 Department of General Surgery, Affiliated Hospital of Nantong University, Nantong, Jiangsu Province 226001, P. R. China; 3 Visitor scholar of Wake Forest Institute for Regenerative Medicine, Wake Forest University School of Medicine, Winston-Salem, NC 27101, USA

**Keywords:** CSC, EMT, Pancreatic cancer, miR-200a

## Abstract

**Background:**

Pancreatic cancer is one of the most aggressive cancers, and the aggressiveness of pancreatic cancer is in part due to its intrinsic and extrinsic drug resistance characteristics, which are also associated with the acquisition of epithelial-to-mesenchymal transition (EMT). Increasing evidence suggests that EMT-type cells share many biological characteristics with cancer stem-like cells. And miR-200 has been identified as a powerful regulator of EMT.

**Methods:**

Cancer Stem Cells (CSCs) of human pancreatic cancer cell line PANC-1 were processed for CD24, CD44 and ESA multi-colorstaining, and sorted out on a BD FACS Aria II machine. RT-qPCR was performed using the miScript PCR Kit to assay the expression of miR-200 family. In order to find the role of miR-200a in the process of EMT, miR-200a mimic was transfected to CSCs.

**Results:**

Pancreatic cancer cells with EMT phenotype displayed stem-like cell features characterized by the expression of cell surface markers CD24, CD44 and epithelial-specific antigen (ESA), which was associated with decreased expression of miR-200a. Moreover, overexpression of miR-200a was resulted in down-regulation of N-cadherin, ZEB1 and vimentin, but up-regulation of E-cadherin. In addition, miR-200a overexpression inhibited cell migration and invasion in CSCs.

**Conclusion:**

In our study, we found that miR-200a played an important role in linking the characteristics of cancer stem-like cells with EMT-like cell signatures in pancreatic cancer. Selective elimination of cancer stem-like cells by reversing the EMT phenotype to mesenchymal-to-epithelial transition (MET) phenotype using novel agents would be useful for prevention and/or treatment of pancreatic cancer.

## Background

Pancreatic cancer is one of the most aggressive cancers, which is usually diagnosed in an advanced state for which there are few or no effective therapies [1]. One of the major hallmarks of pancreatic cancer is its extensive local tumor invasion and early systemic dissemination [2]. Over the past two decades, numerous efforts have been made in improving treatment and survival of pancreatic cancer patients but the outcome has been disappointing [3]. Emerging evidence suggest that the resistance could in fact be due to the enriched existence of tumor initiating cells, also classified as cancer stem-like cells in a tumor mass. The CSCs have the capacity of self-renewaland the potential to regenerate into all types of differentiated cells giving rise to heterogeneous tumor cell populations in a tumor mass, which contributes to tumor aggressiveness [4,5]. The existence of CSCs or cancer stem-like cells in a tumor mass is believed to be responsible for tumor recurrence because of their intrinsic and extrinsic drug-resistance characteristics [3].

Oct4 and Nanog are transcription factors essential for maintaining stem cell phenotypes. Oct4 is a POU domain-containing transcription factor. It is involved in the regulation of cell growth and differentiation in a variety of tissues. Nanog is a homeobox-containing transcription factor. Its overexpression is associated with the pluripotency and self-renewing nature of embryonic stem cells. And Oct4 and Nanog expression is associated with early stages of pancreatic carcinogenesis [6].

It has recently become clear that EMT is associated with drug resistance and cancer cell metastasis [7]. During this process, the expression of E-cadherin is down-regulated, which is a transmembrane protein essential for the stable adherens junctions, and the expression of the mesenchymal molecules vimentin, fibronectin, and/or N-cadherin are up-regulated [8,9]. In pancreatic cancer cells, EMT is also reported to be a crucial step for tumor cell migration and invasion [10]. Recent studies have demonstrated that EMT plays a great role not only in tumor metastasis, but also in tumor recurrence that is believed to be tightly linked with the biology of cancer stem-like cells or cancer-initiating cells. However, the mechanisms by which EMT cells generate the stem-like cells remain to be elucidated [11-13]. Importantly, emerging evidence implicated the critical role of microRNAs because they are key regulatory molecules in biological and pathologic processes including EMT [7]. MicroRNAs are small and non-coding RNA molecules that can regulate gene expression by interacting with multiple mRNAs and inducing either degradation of mRNA or inhibition of their translation to functional proteins [7,14]. Members of the miR-200 family are downregulated in human cancer cells and tumors due to aberrant epigenetic gene silencing and play a critical role in the suppression of EMT, tumor cell adhesion, migration, invasion and metastasis, by targeting and repressing the expression of key mRNAs that are involved in EMT (ZEB1 and ZEB2), and participates in a signalling network with the E-cadherin transcriptional repressors ZEB1/deltaEF1 and ZEB2/SIP1, and TGF-β2 that is postulated to facilitate maintenance of stable epithelial or mesenchymal states but also allow reversible switching between these states in response to EMT effectors (such as TGF-β) [15,16]. In ovarian and breast cancer, low expression of miRNA-200 plays important roles in cancer metastasis [14,17,18]. MiR-200 changed the tumor environment, inhibiting the process of EMT and metastasis [19,20]. These findings hypothesizes that the expression of miR-200 in pancreatic cancer cell is correlated with stemness, EMT and metastasis.

Since miR-200 is associated with EMT, which is believed to be associated with cancer stem cells or cancer stem-like cells, we investigated the effects of miR-200 family on pancreatic CSC functions in this study. We identified a highly tumorigenic subpopulation of pancreatic cancer cells expressing the cell surface markers CD24, CD44 and ESA in pancreatic adenocarcinoma cell line PANC-1. And CD24^+^CD44^+^ESA^+^ cells in PANC-1 were sorted by BD FACS Aria II for further study. Then, we analysed the miR-200 family and transcription factors Oct4 and Nanog expression in CSCs of pancreatic cancer cell line PANC-1, and determined their relationships with EMT markers and repressors of E-cadherin transcription. In order to study the role of miR-200a for EMT in CSCs, miR-200a mimic was transformed into CSCs. In addition, invasion and metastasis were determined in CSCs and transformed CSCs.

## Methods

### Cell culture

Human pancreatic adenocarcinoma cell line, PANC-1 (Chinese Academy of Sciences, Shanghai, P.R. China) was cultured in DMEM supplemented with 10% fetal bovine serum (FBS), 100 U/ml penicillin G, and 100 Ug/ml streptomycin. After sorting, cancer CSCs from the cell line were cultured in either Celprogen’s pancreatic CSC medium or DMEM-F12 supplemented with 1% N2 Supplement (Invitrogen), 2% B27 Supple-ment (Invitrogen), 20 ng/ml human platelet growth factor (Sigma-Aldrich), 100 ng/ml epidermal growth factor (Invitrogen) and 1% antibiotic-antimycotic (Invitrogen) at 37°C in a humidified atmosphere of 95% air and 5% CO_2_.

### Flow cytometry

Dissociated cells were counted and transferred to a 5 ml tube, washed twice with PBS, counted and resuspended in PBS at 1 × 10^6^ cell/100 μl. Then, the antibodies APC anti-human CD44 (Becton Dickinson, USA), PE anti-human CD24 (Becton Dickinson, USA) and FITC anti-human ESA (Becton Dickinson, USA) (each at a dilution of 1: 40) were added and incubated for 20 min on ice in dark. The respective isotype control antibodies were used at the same concentrations according to the manufacturer’s instructions. After washing twice with PBS, samples were resuspended in 500 μl PBS and analyzed on a flow cytometer (FACSAriaII, USA). Side-scatter and forward-scatter profiles were used to eliminate cell doublets. Cells were routinely sorted twice, and the cells were reanalyzed for purity, which typically was > 97%.Data were analyzed with BD FACS Diva software.

### MiR-200a mimic transfection

The PANC-1 cells and CD24^+^CD44^+^ESA^+^ populations of PANC-1 cells were plated in 6 well plates and incubated overnight. Cells were transfected with either control (5′-UUGUACUACACAAAAGUACUG-3′) or miR-200a (forward 5′-UAACACUGUCUGGUAACGAUGU-3′, reverse 5′-AUCGUUACCAGACAGUGUUAUU-3′) mimic at a final concentration of 25 nM using EntransterTM-R transfection reagent (Engreen). After 6 h of transfection the medium was changed to avoid cell death during transfection. Control and miR-200a mimic were purchased from Invitrogen.

### RNA extraction and real-time reverse transcriptase-PCR

Total RNA was extracted using Trizol (Invitrogen) according to the manufacturer’s instructions. For mRNA analysis, real-time PCR was performed using Power SYBR_ green PCR master mix (Applied Biosystems) on an ABI 7500 series PCR machine Applied Biosystems, and data were normalized to GAPDH expression and further normalized to the negative control unless otherwise indicated. Custom primers for E-cadherin, N-cadherin, vimentin and ZEB1, Oct4 and Nanog were synthesized by Ruian biotech. E-cadherin forward primer 5′-GACCGAGAGAGTTTCCCTACG-3′, reverse primer 5′-TCAGGCACCTGACCCTTGTA-3′; N-cadherin forward primer 5′-GAGATCCTACTGGACGGTTCG-3′, reverse primer 5′-TCTTGGCGAATGATCTTAGGA-3′; vimentin forward primer 5′-CCTTGAACGCAAAGTGGAATC-3′, reverse primer 5′-TGAGGTCAGGCTTGGAAACAT-3′; ZEB1 forward primer 5′-GGAATGTATGCTTGTGATTTGTG-3′, reverse primer 5′-CTCTCTTACAGTAGGAGTAGCGATG-3′; Oct4 forward primer 5′-ATTCAGCCAAACGACCATCT-3′, reverse primer 5′-TCTCACTCGGTTCTCGATACTG-3′; Nanog forward primer 5′-AAGAACTCTCCAACATCCTGAAC-3′, reverse primer 5′-CCTTCTGCGTCACACCATT-3′.

### miR expression analysis

Total RNA was reverse transcribed using the miScript Reverse Transcription Kit (Qiagen, Valencia, CA). RT-qPCR was performed using the miScript PCR Kit (Qiagen). Experiments were normalized to U6. Results were reported as RQ with respect to a calibrator sample using the 2-ΔΔCt method. MiR-200a, miR-200b, miR-200c and U6 kits were purchased from Biomics biotech (NanTong).

### Western blot analysis

Cells were lysed in RIPA lysis buffer and the protein concentration was determined. Total proteins were fractionated using SDS-PAGE and transferred onto a polyvinylidene fluoride membrane. The membranes were blocked in 5% skim milk in TBST buffer containing 0.1% Tween 20 and then incubated with indicated primary antibodies (Rabbit anti-N-cadherin, 1: 1000, Millipore; Mouse anti-Vimentin, 1: 500, Millipore; Rabbit anti-E-cadherin, 1: 1000, Abgent; Rabbit Anti-ZEB1, 1: 1000, Cell Signaling) for 2 h at room temperature. HRP-conjugated secondary antibodies (HRP Goat anti-Rabbit IgG Antibody, 1: 5000, Abgent; HRP Goat anti-Mouse IgG Antibody, 1: 5000, Abgent) were incubated at room temperature (RT) for 1 h and detected using the enhanced chemiluminesence detection system.Total protein was extracted from untreated and the cells treated by transfecting miR-200a and subjected to western blot analysis as described to evaluate the expression of E-cadherin, N-cadherin, ZEB1 and vimentin. The data was adjusted against loading control using β-actin.

### Transwell migration assay

For transwell migration assays, 1 × 10^5^ pancreatic CSCs were plated in the top chamber onto the noncoated membrane (24-well insert; pore size, 8 mm; Corning Costar) and allowed to migrate toward serum-containing medium in the lower chamber. Cells were fixed after 48 hours of incubation with methanol and stained with 0.1% crystal violet (2 mg/mL, Sigma-Aldrich). The number of cells invading through the membrane was counted under a light microscope (three random fields per well).

### Transwell invasion assay

For invasion assay, 1 × 10^5^ cells were plated in the top chamber onto the Matrigel coated Membrane (24-well insert; pore size, 8 μm; Corning Costar). Each well was coated freshly with Matrigel (60 mg; BD Bioscience) before the invasion assay. Cells were plated in medium without serum or growth factors, and medium supplemented with serum was used as a chemo-attractant in the lower chamber. The cells were incubated for 48 hours and cells that did not invade through the pores were removed by a cotton swab. Cells on the lower surface of the membrane were fixed with methanol and stained with crystal violet. Thenumber of cells invading through the membrane was counted under a light microscope (three random fields per well).

### Statistical analyses

All values were expressed as means ± S.E.M. The statistical significance of differences among groups was determined by a one-way analysis of variance (ANOVA) followed by the Tukey’s post hoc multiple comparison tests. A P < 0.05 was considered significant. Each experiment consisted of at least three replicates per condition. All statistical analyses were conducted with a STATA 7.0 software package (Stata Corp, College Station, TX) experiments.

## Results

### Isolation and characterization of human pancreatic CSCs

**Figure 1:**
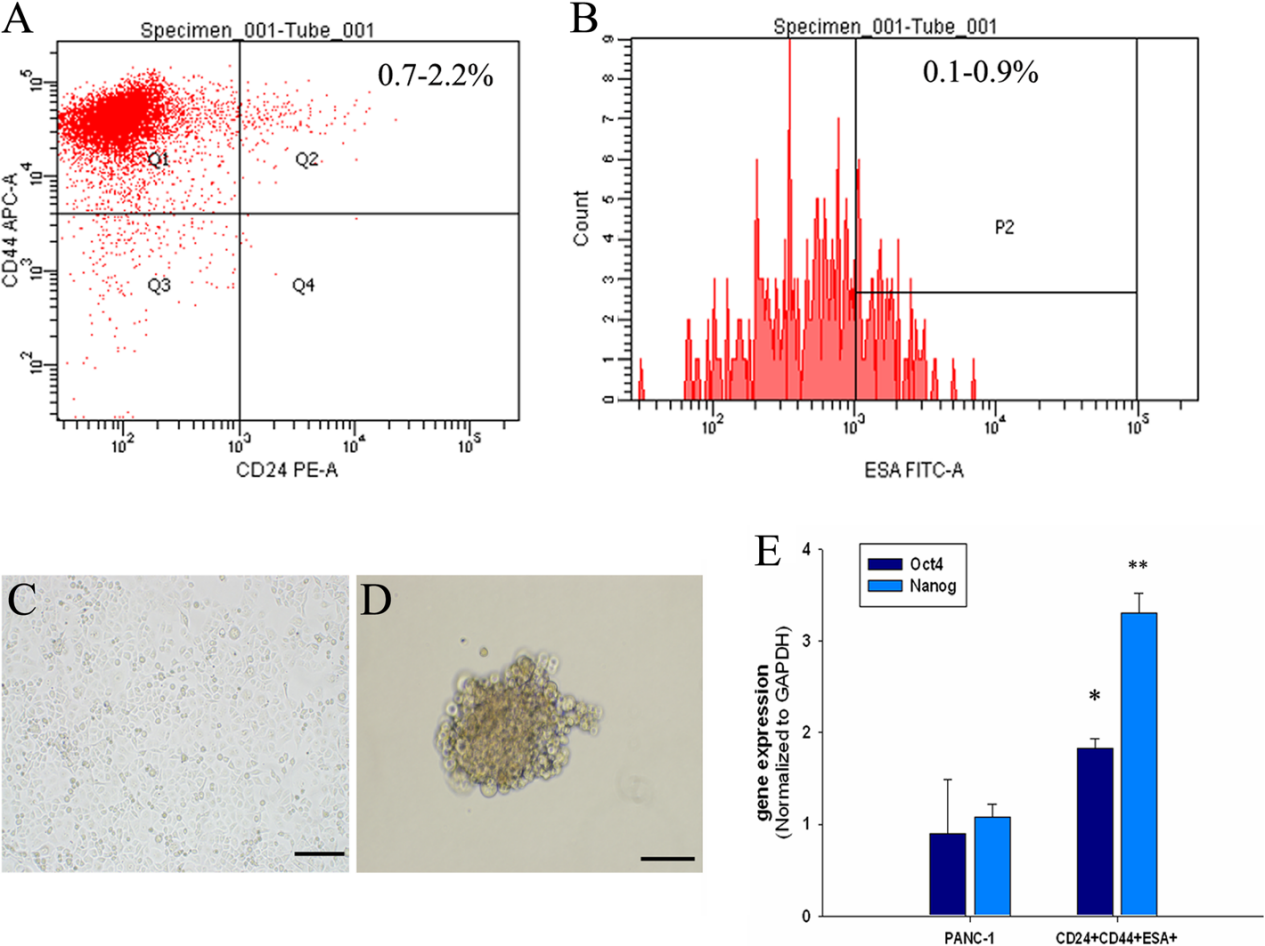
**Presence of CD44, CD24 and ESA on cell surface in pancreatic carcinoma cell line PANC-1.** Flow cytometric analysis was used to determine the presence of CD44, CD24, and ESA on the cell surface of pancreatic adenocarcinoma cell line PANC-1. **(A)** The presence of CD44^+^CD24^+^ population (Q2) of PANC-1 cell line. **(B)** The presence of CD44^+^CD24^+^ESA^+^ population (P2) of PANC-1 cell line. The morphology of PANC-1 cell line **(C)** and CSCs **(D)** (Scale bar = 50 μm). **(E)**The expressions of Oct4 and Nanog were measured by real-time PCR in PANC-1 and CSCs. Data represent mean ± SD. * = significantly different from PANC-1, P < 0.05, ** = significantly different from PANC-1, P < 0.001.

Flow cytometric analysis was used to determine the presence of CD44, CD24 and ESA on the cell surface of the pancreatic adenocarcinoma cell lines PANC-1 (Figure 1A and B). In PANC-1, 0.7%-2.2% of cells were CD44^+^CD24^+^, and 0.1%-0.9% of cells were CD44^+^CD24^+^ESA^+^. The morphology of PANC-1 cell line and CD44^+^CD24^+^ESA^+^ CSCs were shown in (Figure 1C and D). CD44^+^CD24^+^ESA^+^ CSCs formed spheroid in CSC medium × 200 magnification (Figure 1D). Nanog and Oct4 are required for the maintenance of pluripotency in embryonic stem cells and induce cancer stem cell–like properties [21]. To determine stem cell characteristics of CD44^+^CD24^+^ESA^+^ cells, real time-PCR was used to determine the expressions of transcription factors Oct4 and Nanog in CSCs. The results showed that Oct4 and Nanog expressions were significantly increased in CSCs (Figure 1E).

### MIR-200a and EMT markers expression in CSC of PANC-1

**Figure 2:**
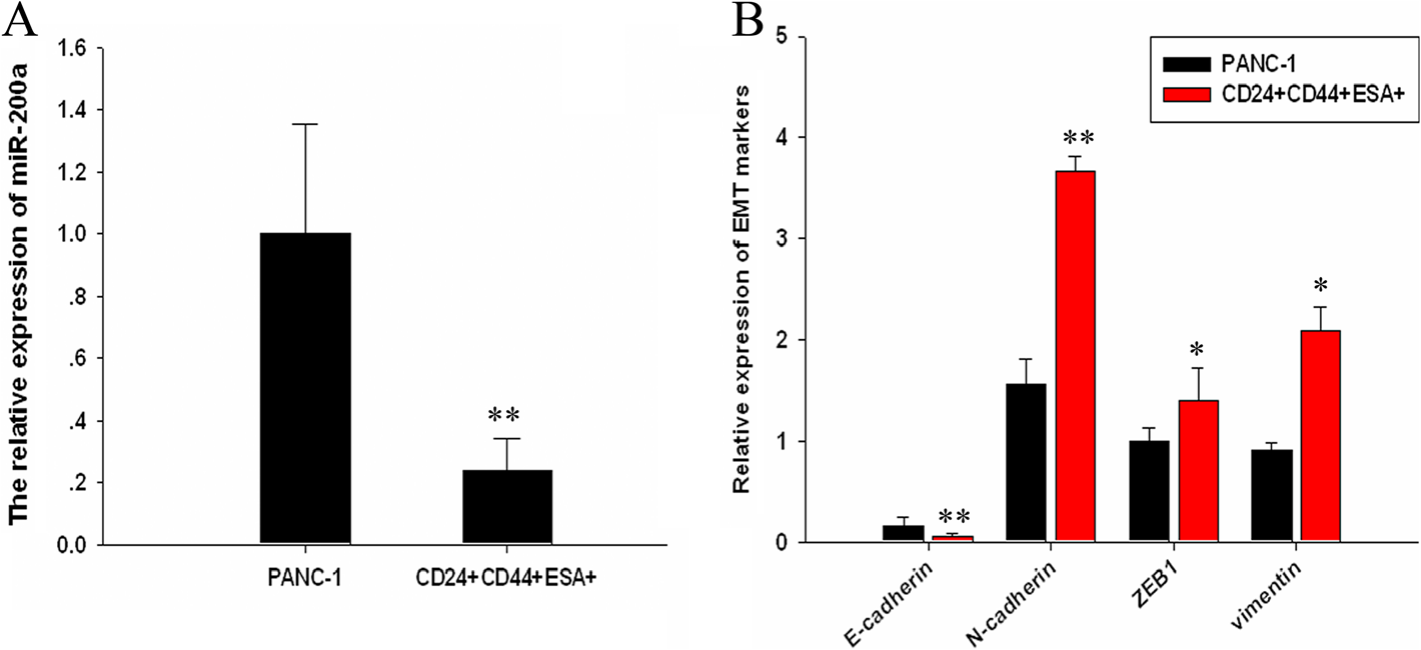
**miR-200a and EMT markers expression in CSCs of PANC-1. (A)** Real-time PCR analyzed the expression of miR-200a in PANC-1 and CSCs. **(B)** Real-time PCR analyzed the expression of E-cadherin, N-cadherin, ZEB1 and vimentin in PANC-1 and CSCs. Data represent mean ± SD. * = significantly different from PANC-1, P < 0.05, ** = significantly different from PANC-1, P < 0.001.

MiR-200 family is known as tumor suppressor and they are usually down-regulated in EMT in some cancer stem cells, such as in prostate cancer and breast cancer [22,23]. MiR-200/ZEB2 pathway in mesenchymal-to-epithelial transition and induced pluripotent stem cell generation [24]. We determined the expression levels of miR-200a, miR-200b and miR-200c in PANC-1 cell line and CSCs by real time-PCR. Real time-PCR result showed that miR-200a were significantly downregulated in CSCs (Figure 2A), while miR-200b and miR-200c expressions were similar with PANC-1 cell line (data was not shown). EMT is accompanied by E-cadherin down-regulation and N-cadherin up-regulation. We found the expressions of N-cadherin, vimentin and ZEB1 were up-regulated, while the expression of E-cadherin exhibited down-regulation at mRNA levels in CSCs of pancreatic cancer cell line PANC-1. These results suggested that CSCs showed EMT characteristics which was defined by the expression of specific miRNA and its target genes (Figure 2B).

### MIR-200a regulated EMT of CSCs in pancreatic cancer

**Figure 3:**
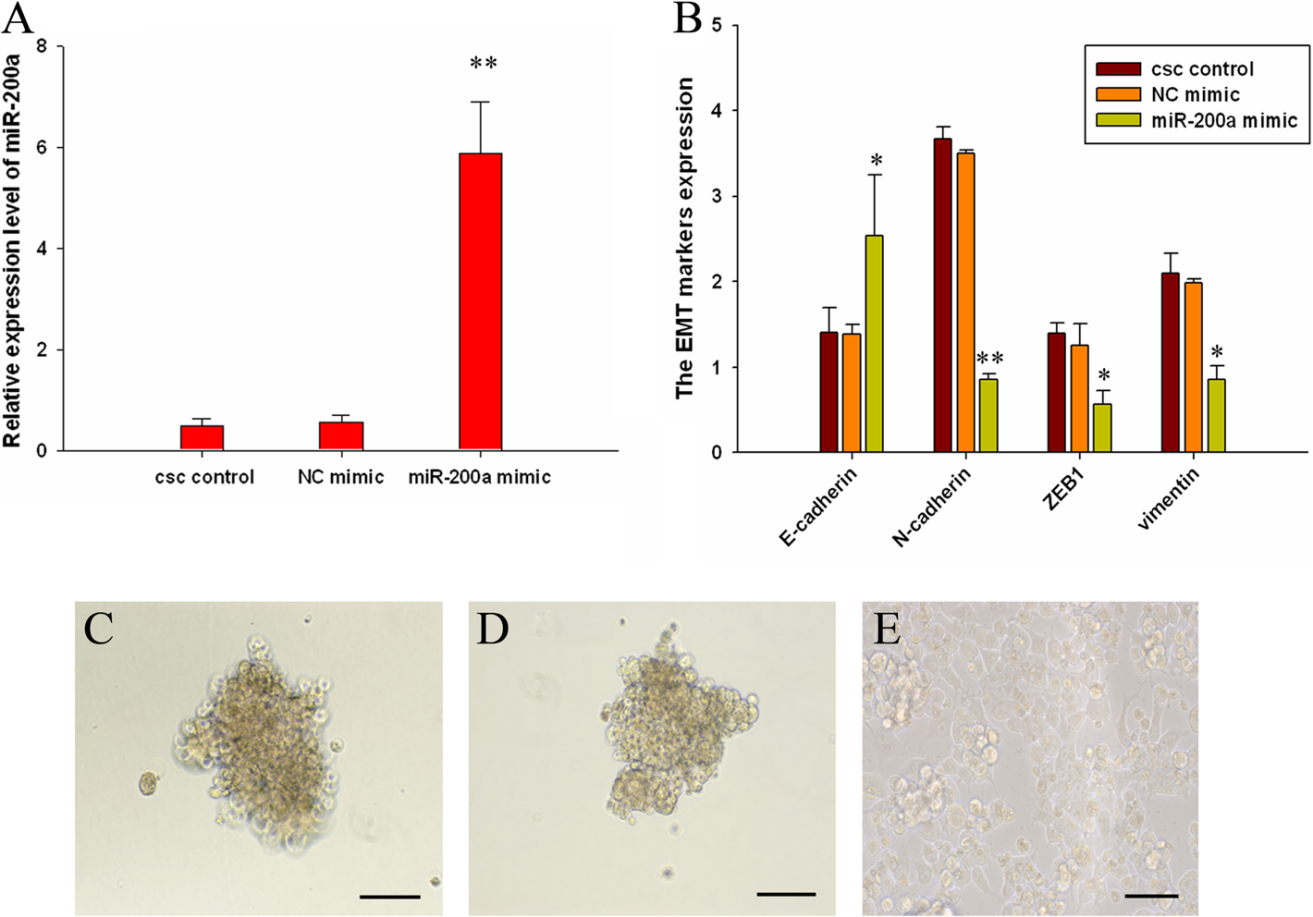
**miR-200a regulated EMT of CSCs in pancreatic cancer. (A)** MiR-200a mimic was efficient in CSCs. **(B)** Overexpression of miR-200a in CSCs of PANC-1 resulted in up-regulation of E-cadherin and down-regulated expression of N-cadherin, ZEB1 and vimentin as assessed by Real-Time PCR. **(C-E)** The morphology of CSCs changed from sphericity to epithelial-like appearance after miR-200a transfection. **(C)** The morphology of CSCs control, **(D)** CSCs transfected by control mimic and **(E)** CSCs transfected by miR-200a mimic (Scale bar = 50 μm). Data represent mean ± SD. * = significantly different from control, P < 0.05, ** = significantly different from control, P < 0.001. Data represent mean ± SD.

**Figure 4:**
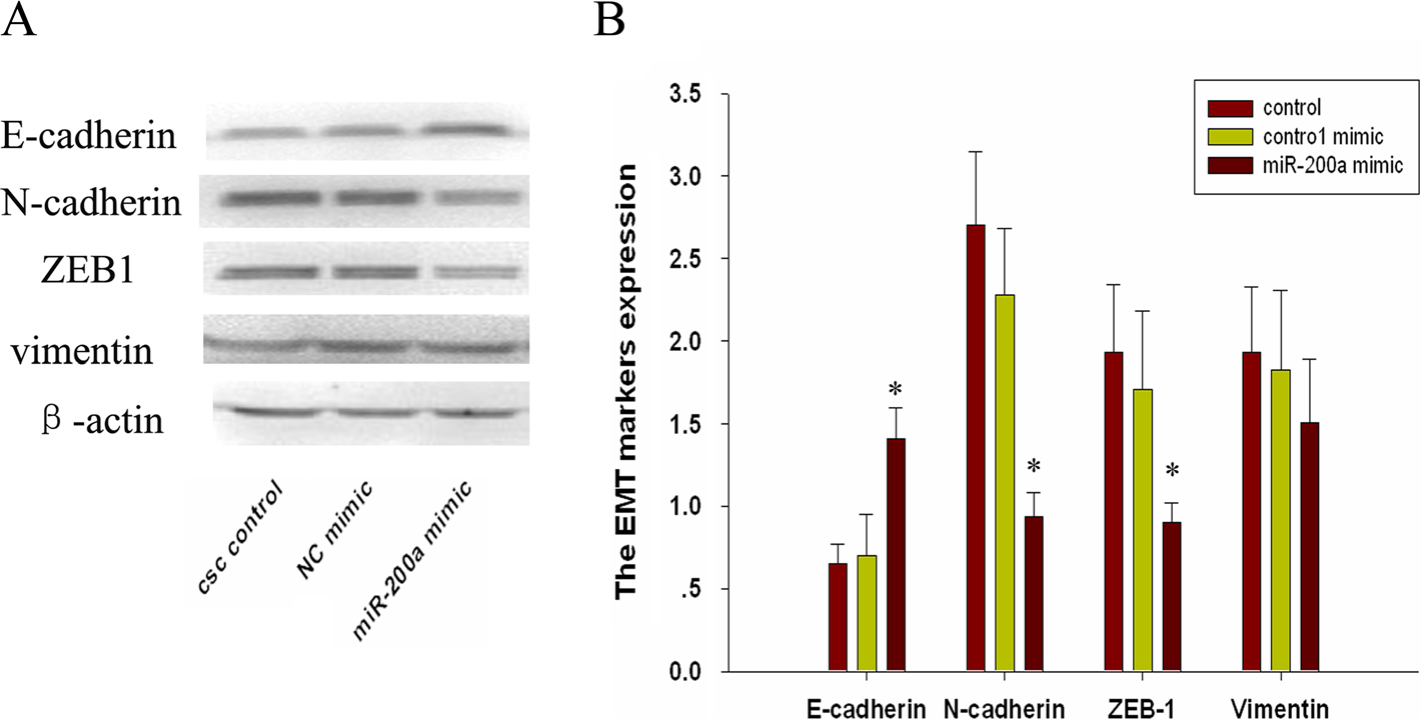
**miR-200a induced MET in human pancreatic CSCs. (A)** Western blot analysis showed the expression of E-cadherin was up-regulated and the expression of ZEB1 and N-cadherin was down-regulated in CSCs transfected by miR-200a mimic. **(B)** Quantification of immunoblot data. Data represent mean ± SD. * = significantly different from control, P < 0.05, ** = significantly different from control, P < 0.001.

Members of miR-200 family are known to be tumor suppressors, which are usually downregulated in some tumors. Down-regulation of miR-200 by siRNA technique has been shown to be associated with EMT phenotype while overexpression of miR-200a can result in the reversal of EMT phenotype [22]. To investigate the role of miR-200a in the reversal of EMT phenotype of CSCS, we transfected miR-200a mimics or non-specific control miRNA mimic (NC mimic) into CSCs (Figure 3A). We found that the overexpression of miR-200a in the CSCs resulted in the up-regulation of epithelial marker E-cadherin and down-regulation of mesenchymal markers ZEB1, N-cadherin and Vimentin at mRNA level (Figure 3B). These results suggest that the loss of miR-200a is critical for the acquisition of EMT characteristics and that the overexpression of miR-200a could reverse the EMT phenotype of CSCs. After transfection, the morphology of miR-200a transfected CSCs was partially changed from elongated fibroblastoid to epithelial cobblestone-like appearance, and the cells appeared to grow in close contact with each other (× 200 magnification) (Figure 3C-E). By westernblot analysis, we found that the overexpression of miR-200a in the CSCs resulted in the up-regulation of epithelial marker E-cadherin and down-regulation of mesenchymal markers ZEB1 and N-cadherin, but not Vimentin at protein level (Figure 4A and 4B).

### MiR-200a overexpression decreased the ability of invasion and migration of CSCs

**Figure 5:**
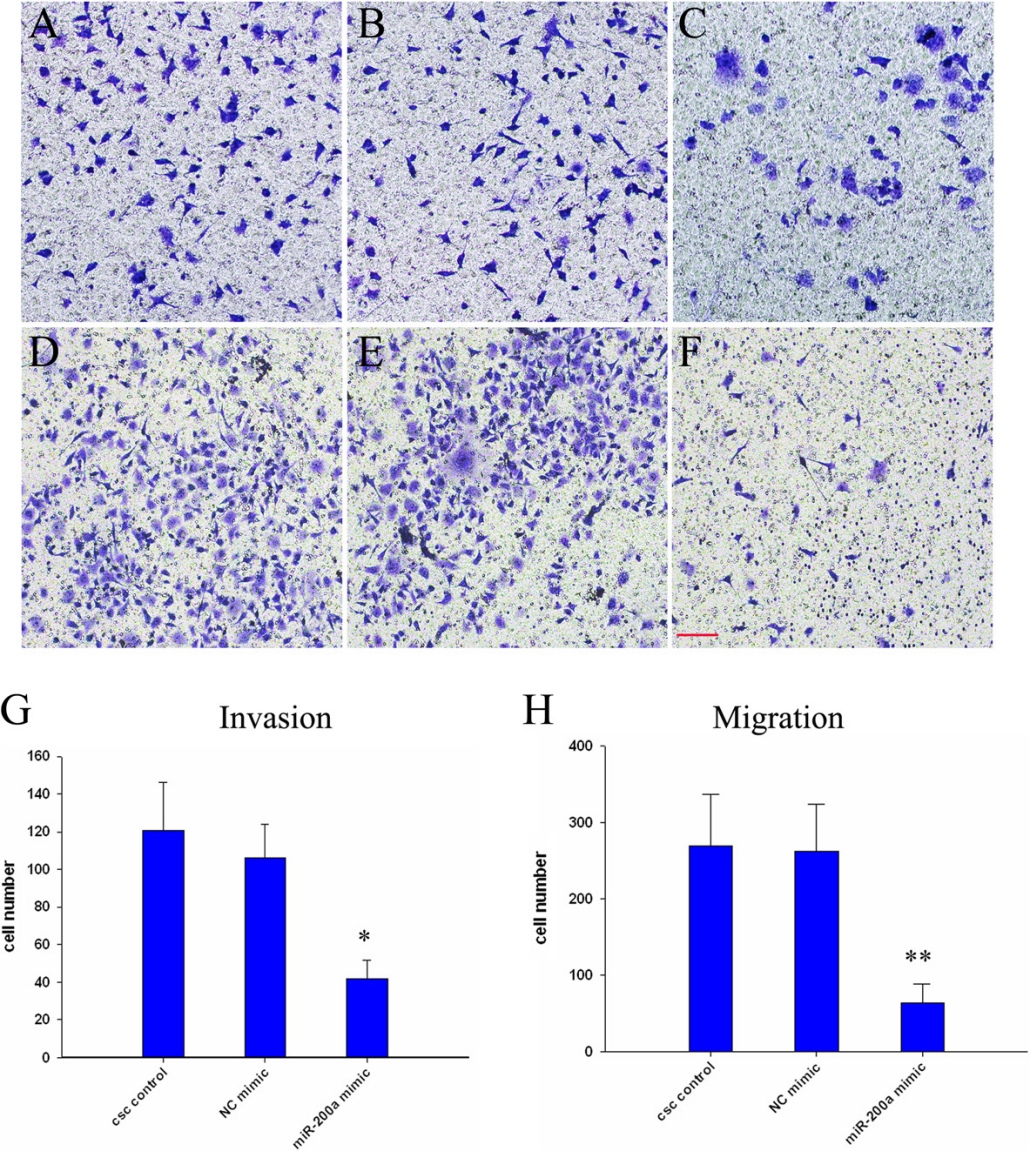
**miR-200 reduces invasion and migration in human pancreatic cancer.** The number of cells invasion and migration in the miR-200a mimic treated groups were decresed **(A-F)**. **(A and D)** CSCs control, **(B and E)** NC mimic, **(C and F)** miR-200a mimic, **(A-C)** the number of cells invasion and **(D-F)** the number of cells migration. **(G and H)** Quantification of invasion and migration data. Data represent mean ± SD. * = significantly different from control, P < 0.05, ** = significantly different from control, P < 0.001 (Scale bar = 50 μm).

EMT induction in cancer cells results in the acquisition of invasive and metastatic properties [25]. To evaluate the invasive and migration potential of miR-200 overexpression CSCs, an assay was performed using Transwell inserts. The results showed that the number of CSCs transfected with miR-200a mimic which invaded and migrated to the lower side of the membrane was significantly decreased than CSCs or NC mimic by about 0.33 fold in invasion and 0.22 fold in migration, respectively (Figure 5). These results clearly suggested that overexpression of miR-200a inhibits migration and invasion of CSC cells through the reversal of EMT to MET phenotype.

## Discussion

Pancreatic cancer is the fourth to fifth leading cause of cancer-related death in western societies with an average overall 5-year survival of less than 5% and a median survival period of less than 6 months [26]. CSCs are a small subpopulation of cells capable of self-renewal and differentiation and have been identified in a variety of tumors. CSCs may be responsible for tumor initiation, progression, metastasis and resistance to therapy. The expression patterns of the stem cell surface markers CD44, CD24 and ESA in pancreatic adenocarcinoma cell lines are diverse and not all CSCs sorted from pancreatic adenocarcinoma cell lines develop cell spheres [27], PANC-1 is a common pancreatic adenocarcinoma cell line used for study of CSCs [28-32]. In this study, we sorted CD44^+^CD24^+^ESA^+^ CSCs in pancreatic adenocarcinoma cell line PANC-1. Stem cell features are controlled by a small group of transcription factors, like Nanog and Oct4 [33]. Coexpression of Oct4 and Nanog is associated with pancreatic carcinogenesis and is negatively correlated with the survival prognosis of oral squamous cell carcinoma patients [34]. Oct4 and Nanog induce cancer stem cell–like properties and have correlations with EMT [35]. Our study also demonstrates that the expressions of Oct4 and Nanog were increased in CSCs of PANC-1.

Emerging evidence suggests the role of microRNA (miRNA) in many biological processes. Among many miRNAs, miR-200 family is known as tumor suppressor and they are usually down-regulated in some tumors including prostate cancer and the loss of expression of miR-200 family contributes to the acquisition of EMT phenotype and drug resistance. Down-regulation of miR-200 by siRNA technique has been shown to be associated with EMT phenotype while reexpression of miR-200 can result in the reversal of EMT phenotype. We determined the expression levels of miR-200a, miR-200b and miR-200c in CSCs of PANC-1 by real time RT-PCR. Among the family of miR-200, only miR-200a expression was dramatically decreased.

EMT induction in cancer cells results in the acquisition of invasive and metastatic properties. Recent reports indicate that the emergence of CSCs occurs in part as a result of EMT, for example, through cues from tumor stromal components. CSCs and EMT-type cells, which shares molecular characteristics with CSCs, have been believed to play critical roles in drug resistance and early cancer metastasis as demonstrated in several human malignancies including pancreatic cancer. Interestingly, recent studies have also shown that miR-200 family could regulate the processes of EMT by targeting E-box binding protein ZEB1 and ZEB2 [36]. Thus, the discovery of molecular knowledge of drug resistance and metastasis in relation to miR-200 family, CSCs and EMT in pancreatic cancer are becoming an important area of research, and such knowledge is likely to be helpful in the discovery of newer drugs as well as designing novel therapeutic strategies for the treatment and/or prevention of pancreatic cancer with better outcome. E-cadherin, occludin and cytokeratin are downregulated during EMT, while N-cadherin, vimentin, fibronectin, SNAI1/SAIL, SNAI2/SLUG, ZEB2/SIP1, and TWIST1 are upregulated class switch from E-cadherin to Ncadherin results in the loss of epithelial phenotype and the acquisition of mesenchymal phenotype. Transcriptional repression of E-cadherin gene or functional repression of E-cadherin protein is the critical step for EMT. Upregulation of EMT regulators is associated with more malignant phenotypes in a variety of human cancer, such as gastric cancer, pancreatic cancer, breast cancer, and ovarian cancer [37]. In the present study, miR-200a inhibits epithelial-mesenchymal transition by inhibiting the expression of vimentin, N-cadherin and ZEB1, and also retards CSCs migration and invasion. The inhibition of EMT markers by these agents suggests that they could inhibit early metastasis of pancreatic CSCs.

The EMT is an embryonic key developmental program that is often activated during cancer invasion and metastasis. It is a process by which cells undergo a morphological switch from the epithelial polarized phenotype to the mesenchymal fibroblastoid phenotype. Many signalling pathways have contributed to the induction of EMT, including TGFβ-1, Wnt, Hedgehog, Notch, and nuclear factor-kappa B (NF-kB) [38-42]. Kyoung-Ok Hong et al. have shown that activation of PI3K/Akt axis is one of the key mechanisms in the process of EMT and it seems that its inhibition by treatment with phosphatidylinositol ether lipid analogues (PIA) may regulate the reverse process MET leading to the re-expression of both E-cadherin and β-catenin, and reducing expression of vimentin, mesenchymal marker, in oral squamous lines carcinoma stabilized [43]. Further stuty was needed to probe the signalling pathways participatived in MET of CSCs.

## Conclusion

In summary, we identified a highly tumorigenic subpopulation of pancreatic cancer cells expressing the cell surface markers CD24, CD44 and ESA in pancreatic adenocarcinoma cell line PANC-1. MiR-200a played important roles in the MET process of CSCs by changing EMT markers expressions and cell migration and invasion. Thus, further studies are needed to elucidate how miR-200a eliminates the stem-like cells by reversing EMT phenotypic cells and to assess whether miR-200a could be useful for the prevention of pancreatic cancer tumorigenesis and metastasis.

## Competing interests

The authors declare that they have no competing interests.

## Authors’ contributions

YL and ZW conceived and supervised the study. JL, HZ, XF and SZ performed the experiments. YW and QG analyzed and interpreted the data. XL and MZ drafted and revised the manuscript. LW and YH provided technical support. All authors read and approved the final manuscript.

## Pre-publication history

The pre-publication history for this paper can be accessed here:

http://www.biomedcentral.com/1471-2407/14/85/prepub
